# Case Report: The First Reported Concurrence of Wilson Disease and Bilateral Retinitis Pigmentosa

**DOI:** 10.3389/fmed.2022.877752

**Published:** 2022-04-28

**Authors:** Zifan Ye, Xiuhua Jia, Xin Liu, Qi Zhang, Kaijun Wang, Min Chen

**Affiliations:** ^1^Eye Center of the 2nd Affiliated Hospital, School of Medicine, Zhejiang University, Hangzhou, China; ^2^Zhejiang Provincial Key Lab of Ophthalmology, Hangzhou, China; ^3^Department of Ophthalmology, The 3rd Affiliated Hospital of Sun Yat-sen University, Guangzhou, China

**Keywords:** Wilson disease, retinitis pigmentosa, concurrence, gene mutation, abnormal copper metabolism

## Abstract

**Background:**

Wilson disease (WD) and retinitis pigmentosa (RP) are common genetic disorders in clinical practice, however, the concurrence of WD and RP has never been reported before. WD occurs due to mutations that cause copper metabolic abnormalities; in turn, change in copper metabolism has been suggested to be related with RP. Here, we report the first case of concurrent WD and bilateral RP, and investigate possible pathogenesis to illuminate whether the two genetic disorders are causality or coincidence.

**Case Presentation:**

The patient was a 43-year-old Chinese female diagnosed with WD 12 years ago. She had suffered from night blindness since childhood and faced diminution of bilateral vision within 10 years, for which she was referred to our Eye Center during hospitalization for routine copper excretion treatment. The ceruloplasmin, skull magnetic resonance imaging (MRI), and abdominal ultrasound results accorded with hepatolenticular degeneration. Ocular examinations revealed corneal Kayser-Fleischer (K-F) ring, sunflower-like cataract, retinal osteocyte-like pigmentation, bilateral atrophy of outer retina, cystoid macular edema (CME), and tubular vision in both eyes. Phacoemulsification combined with intraocular lens implantation was performed in the right and left eye, but there was limited improvement in her visual acuity. Whole exome sequencing (WES) detected a deleterious homozygous mutation in the *ATP7B* gene related to WD, and a homozygous mutation in the *CNGA1* gene very likely to cause RP.

**Conclusions:**

We reported the first case of concurrent WD and RP. WES detected two pathogenic gene mutations, *ATP7B* and *CNGA1*. Though we cannot completely rule out a causal effect of WD-related abnormal copper metabolism with RP, we speculate that the two gene mutations lead to the coincidence of the two genetic disorders, respectively.

## Introduction

Wilson disease (WD) is an autosomal-recessive disorder caused by mutations in the *ATP7B* gene, leading to abnormal copper metabolism. *ATP7B* encodes the copper transporter in the reverse Golgi network structure of liver cells, which is indispensable for biliary copper secretion and the synthesis of proceruloplasmin with copper atoms (1). Ceruloplasmin, the synthetic product, is then secreted into the blood, mediating the transport of excess copper in cytoplasmic vesicles to prolysosomal vesicles and thereby discharging copper into the bile (1). The obstruction of copper excretion results in the accumulation of copper in the circulatory system and organs, especially the liver, central nervous system (CNS), and eyes. The age of WD onset varies from early childhood to young adulthood, though there are cases of late-onset WD (2). Clinical manifestations can differ according to different age groups. For instance, children are more likely to show hepatic symptoms (3), while older patients (15–17 years old and above) present neurological manifestations more often, including dysarthria, gait abnormalities, dystonia, tremor, and Parkinson's disease (4). The typical ocular manifestations of WD include Kayser-Fleischer (K-F) ring and sunflower-like cataract.

In turn, retinitis pigmentosa (RP) is a group of retina-degenerative diseases characterized by progressive degeneration of retinal pigmented epithelium (RPE) and photoreceptors. It affects ~1 in every 4,000 persons on a global scale (5), making it one of the most common retinal degenerations that contribute to visual impairment in all age groups. There are 69 genes that have been mapped and identified to cause RP so far.^1^ Various inheritance patterns have also been connected to RP, including sporadic, autosomal-dominant, autosomal-recessive, X-linked, mitochondrial, and digenic (6). Of these, autosomal-recessive inheritance accounts for ~50–60% of all RP patients (7). Rod photoreceptor dysfunction is often the first system to occur, followed by injury to the cone photoreceptor cells. Therefore, night blindness, progressive vision loss, tunnel vision, and complete blindness are typical symptoms of RP. Subretinal injection of adeno-associated virus vector, inserting with RPE65 cDNA, may repair this genetic deficiency and may be the only therapy available and approved for RP (8). Whether copper metabolism was associated with RP remains controversial. As early as in 1976, Gahlot et al. reported that RP may be a condition caused by abnormal copper metabolism (9). While in 1978, Marmor et al. argued against a role for copper metabolism in ordinal retinitis pigmentosa (10).

For the first time, we reported a case with the concurrence of WD and bilateral RP in a patient from a consanguineous family. Whole exome sequencing (WES) detected separate gene mutations for the two genetic disorders.

## Case Presentation

**Figure 1:**
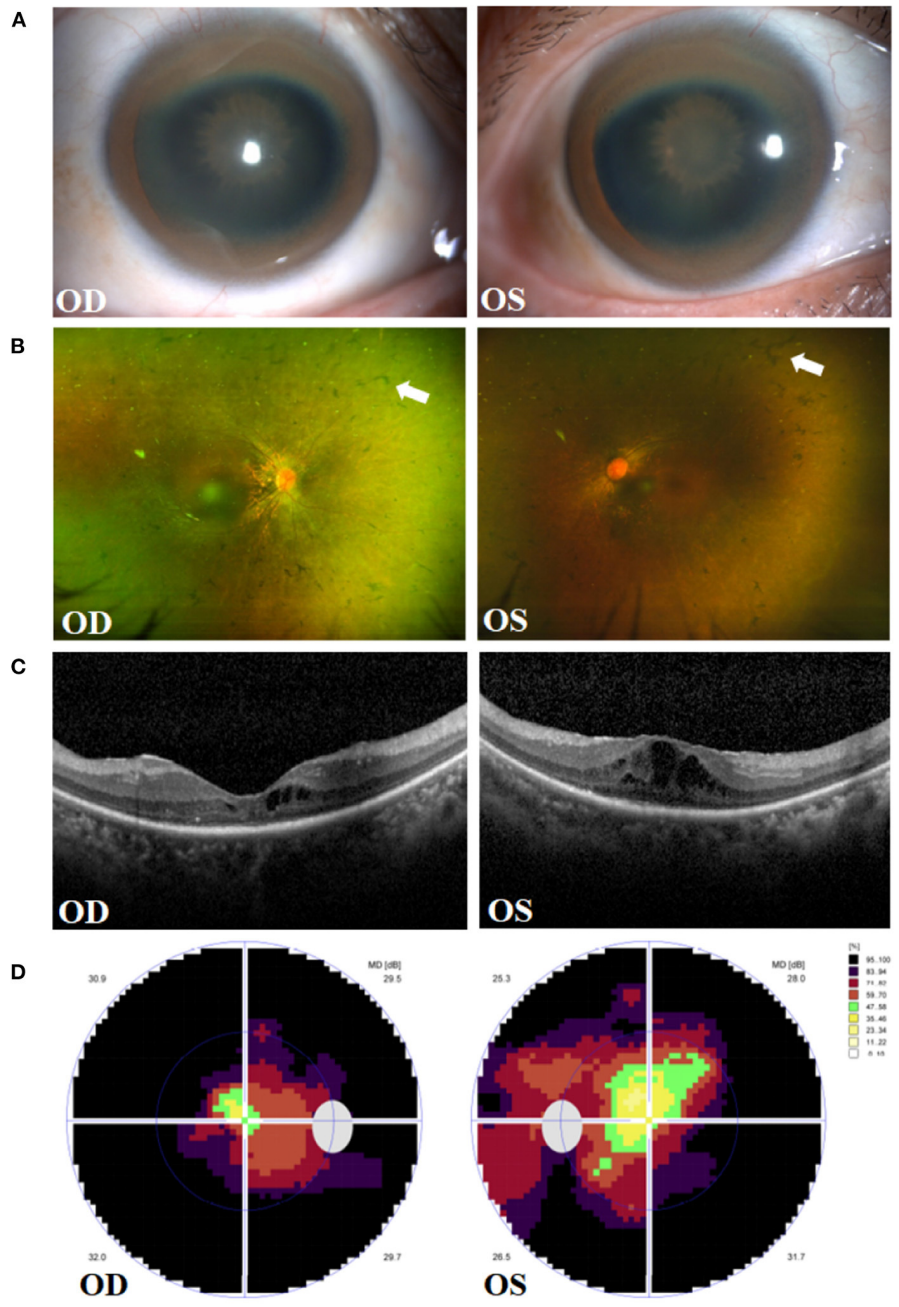
Clinical presentations in the right eye (OD) and left eye (OS) of the patient. **(A)** Manifestation of Kayser-Fleischer (K-F) ring and sunflower-like cataract. **(B)** Fundus images of osteocyte-like pigmentation (white arrows) in bilateral retina. **(C)** Optical coherence tomography **(OCT)** showing outer retina atrophy and cystoid macular edema. **(D)** Vision detection featuring binocular tunnel vision.

A 43-year-old female presented to the Department of Neurology of the Second Affiliated Hospital of Zhejiang University in March 2021. She was diagnosed with WD 12 years ago. Physical examination showed the patient had reduced facial expression and impaired articulation. The patient's left hand mildly trembled when she was asked to raise it flatly. She was also found to have increased extremity muscular tension, upper extremity tendon reflex +++++, and was unable to walk straight. Laboratory tests showed a low ceruloplasmin level (23 mg/L, reference 200–600 mg/L), and no abnormal serum copper concentration was found. Skull MRI showed hepatolenticular degeneration. Abdominal ultrasound revealed liver cirrhosis and splenomegaly. The patient was hospitalized and given sodium dimercaptopropane sulfonate for copper removal, zinc gluconate to inhibit copper metabolism, and supportive treatment such as amantadine and vitamin C supplements. During hospitalization, the patient complained of progressive bilateral vision decrease over 10 years, for which she was referred to our Eye Center. She volunteered that the night blindness began in early childhood, and her parents were close relatives. Her parents and sister were healthy, denying a similar medical history. The patient's best corrected visual acuity (BCVA) at presentation was 0.8 (logarithm of the minimum angle of resolution, logMAR) in both eyes, and intraocular pressure (Non-contact tonometer, Topcon CT-80, Topcon Corporation, Tokyo, Japan) was 10.0 mmHg in the right eye (OD) and 13.5 mmHg in the left eye (OS), respectively. Slit lamp biomicroscopy (SL-D8Z; Topcon Corporation, Tokyo, Japan) revealed bilateral corneal K-F ring and sunflower-like cataract (Figure 1A). Fundus photography (TRC-NW8; Topcon Corporation, Tokyo, Japan) showed thinner retinal blood vessels and retinal osteocyte-like pigmentation in bilateral eyes (Figure 1B). Cystoid macular edema (CME) and outer retina atrophy was observed in both eyes via optical coherence tomography (OCT) (Figure 1C). Visual field examination (Octopus 900, Haag-Streit, USA) revealed binocular tunnel vision (Figure 1D). The patient was subsequently diagnosed with WD combined with binocular RP and complicated cataract.

After informed consent form the patient, phacoemulsification and posterior chamber intraocular lens implantation was performed in the right and left eye, respectively. Postoperatively, topical Tobradex (Tobramycin and Dexamethasone, Alcon) and Pranoprofen (Senju Pharmaceutical Co.Ltd, Japan) eyedrops were prescribed four times a day for anti-inflammatory treatment. Drug therapy for CME was not started before the surgery, and there was no significant change in the central retinal thickness (CRT) of both eyes during the 8-months follow-up. Unfortunately, there was limited improvement in her visual acuity. The BCVA remains 0.8 logMAR for both eyes at her last visit (Supplementary Figure 1).

**Figure 2:**
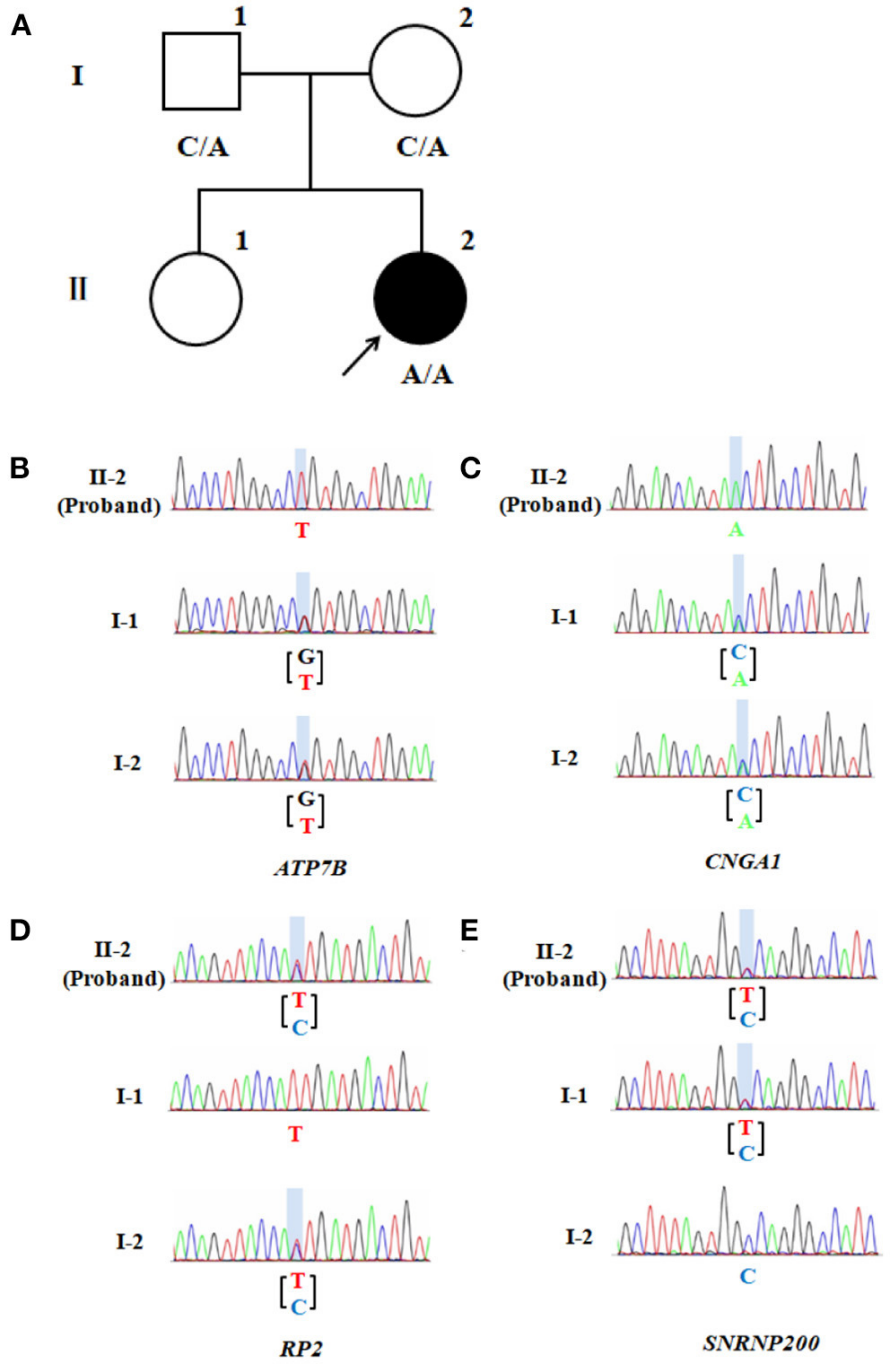
Pedigree of the patient's family. **(A)** *CNGA1* variant family pedigree. Circles represent females and the square represents the male. The filled circle represents the patient with RP. The proband is indicated by a black arrow, while A represents a mutation. **(B)** Partial sequence of *ATP7B* gene locus of the proband (II-2) and the unaffected family members (I-1 and I-2). The columnar graphics indicate the site of the variant. **(C)** Partial sequence of the family's *CNGA1* gene locus. **(D)** Partial sequence of the family's *RP2* gene locus. **(E)** Partial sequence of the family's *SNRNP200* gene locus.

Whole exome sequencing (WES, by Beijing Giantmed medical diagnostics Lab) was performed on the patient and her parents (Figures 2A–E). The results showed an *ATP7B* gene c.G2333T: p.R778L homozygous mutation, *CNGA1* gene c.C453A: p.Y151X homozygous mutation, *RP2* gene c.T248C: p.l83T heterozygous mutation, and *SNRNP200* gene c.C1898T: p.A633V heterozygous mutation in the patient (Supplementary Table 1). Both parents were heterozygous carriers of *ATP7B* and *CNGA1* genes. The mother was of the *RP2* heterozygous genotype, and the father was of the *SNRNP200* heterozygous genotype, which were both found in the patient. However, the parents did not show any WD or RP-related manifestations.

## Discussion

To our knowledge, this is the first report of concurrence of WD and RP in a patient from a consanguineous family. WES detected two separate pathogenic gene mutations: a homozygous mutation in the *ATP7B* gene related to WD, and a homozygous mutation in the *CNGA1* gene associated with RP.

WD is an autosomal-recessive disorder characterized by liver cirrhosis and basal ganglia lesions. Apart from liver and brain, copper also accumulates in other organs, for liver injury is supposed to cause secondary impairment in other tissues. Consequently, the clinical manifestations can include cardiac, renal, dermatic, osteoarticular or endocrinologic conditions (11). Corneal K-F ring and sunflower-like cataract are common ocular manifestations of WD. K-F ring is formed by copper particles deposited in the Descemet membrane of the corneoscleral junction area which has been observed in ~98% of patients with neurological WD symptoms and about 50% of patients with hepatic manifestations (12). Sunflower-like cataract is another ocular sign of WD. Compared with typical cataract leading to markedly reduced visual acuity, sunflower-like cataract seems to have a limited impact on vision (13), as it results from reversible copper deposition under the anterior lens capsule and can diminish after copper removal treatment.

Recent researches have pointed out that WD also affect the retina and optic nerve (14, 15). Especially in those patients who have obvious lesions in CNS, thickness of RNFL is found to have decreased, as is the macular thickness (16). Interestingly, disease duration and forms of neurological or hepatic manifestations do not influence RNFL thickness (17). In addition, the thickness of macula is discovered to get markedly thinner in the inferior quadrant (14). Therefore, retinal degeneration in WD is considered to be a marker of neural lesion and correlate with the degree of nerve injury.

**Figure 3:**
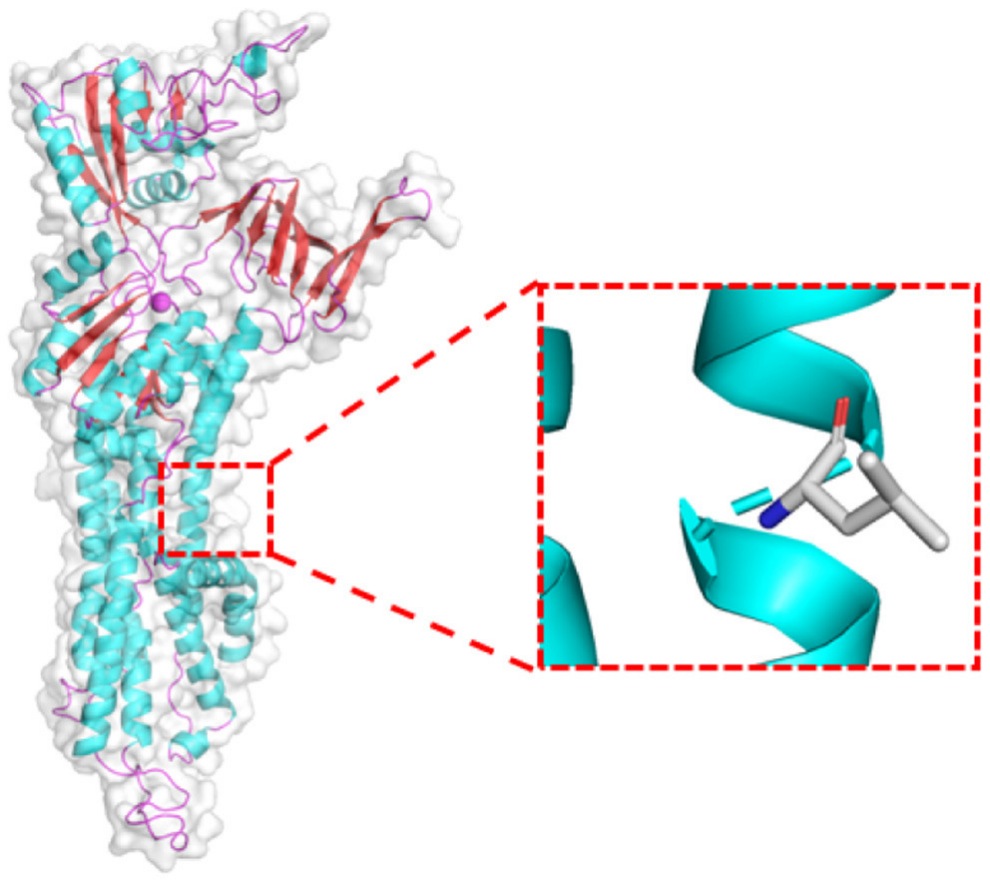
Prediction of the protein structure of the *ATP7B(R778L)* mutant expression product.

*ATP7B* gene mutation has been recognized as the pathogenic genes of WD. The mutations affect the main elimination pathway of liver copper, resulting in copper deposition in various organs, though especially the liver, brain, and eyes. The prevalence rate is 1/10,000 to 1/30,000 worldwide (18) and it is observed to be higher in China (19, 20). Previous research showed that WD in China is seemingly resulted from some relatively common mutations and a large number of rare mutations (20). p.R778L, p.P992L, and p.T935M have been identified to be the top three mutations in China (19). In our case, WES detected the missense c.G2333T: p.R778L variant of *ATP7B*, and the candidate pathogenic mutation site was included in the ClinVar database.^2^ The program Polyphen2 predicted the variant to be probably damaging. According to the American College of Medical Genetics and Genomics (ACMG) guidelines, the mutation was pathogenic and the grade was PM3-very strong. Swiss-Model software^3^ predicted the 3D structure of the pathogenic protein sequence (Figure 3).

Meanwhile, RP is a group of hereditary retinal malnutrition diseases characterized by the progressive degeneration of RPE and photoreceptors (rods and cones). Night blindness, progressive loss of peripheral vision, tunnel vision, and even blindness in advanced stages are typical symptoms (21). CME can be one of its macular conditions and thus may relate to the loss of central vision (22). The prevalence of CME in RP patients varies from 11 to 50% according to different detection methods (23, 24). The etiology of CME in individuals with RP remains uncertain, including (i) breakdown of the blood-retina barrier, (ii) impaired function of the RPE pumping mechanism, (iii) lesions on Müller cells, (iv) anti-retinal antibodies, and (v) vitreous traction (25). In this case, CME was found in both eyes, for which we speculate RP may be responsible.

Monogenic inheritance is responsible for the largest proportion of RP cases, yet the disease is highly heterogeneous. There are 69 genes that have been mapped and identified to cause RP so far,^4^ most of which contribute to non-syndromic RP. Apart from the non-syndromic type, RP also occurs in diseases that affect other sensory nervous systems or multiple tissues, such as Usher syndrome, Bardet-Biedl syndrome, and Cohen syndrome (26).

With the current patient, WES identified three different mutations in genes related to RP. *RP2* and *SNRNP200* have been mapped to RP2 and RP33, respectively, but the pathogenesis remains ambiguous, and their ACMG pathogenic grades are both of “uncertain significance” (*RP2*: PM1 + PM2-supporting + PP3; *SNRNP200*: PM1 + PP3). *CNGA1* is one of the causative genes of autosomal-recessive RP (27), as related to RP 49 (autosomal-recessive). As a member of the cyclic nucleotide-gated cation channel subfamily, *CNGA1* encodes the α-subunit of the rod cGMP-gated channel (28). As an essential protein of the rod photoreceptors in the cascade reaction of light conduction, rod cGMP-gated channel is composed of three CNGA1 subunits and one CNGB1 subunit (29). Besides, CNGA1 is also required for the structure of the outer segment of rod photoreceptors (30). Several mutations to *CNGA1* have been identified to be deleterious and have a causal link to autosomal-recessive RP (31). Homozygous *CNGA1* variant c. C453A: p. Y151X has been reported once (32), though it was detected to be nonsense and has a prevalence of 0.00005 in a crowd. This pathogenic grade suggests the variant to be “pathogenic” for the classification of evidential items assessed by ACMG guidelines shows PVS1 + PM2-supporting + PM3-very strong. Since the current patient's parents did not exhibit RP-related symptoms, we surmise that the homozygous *CNGA1* variant in the patient conformed to gene co-segregation and might be the variant leading to RP. 3D structure of the pathogenic protein sequence cannot be predicted owing to the nonsense mutation that caused excessive deletion of amino acid fragments.

Metal cations are required in numerous biological processes, as in the pathogenesis of RP. For example, the role of zinc cation in the development of RP has been illustrated to be related to the loss of thermostability of the rhodopsin protein and zinc coordination to amino acid residues (33). Severe brain iron deposition is also implicated in the pathogenesis of neurodegenerative disorders and inherited diseases. As a group of progressive extrapyramidal disorders, neurodegeneration with brain iron accumulation (NBIA) can present with RP in child cases which are classified as pantothenate kinase-associated degeneration (PKAN) and this can lead to significant visual impairment (34, 35). In addition, iron metabolic dysfunction, which cause iron accumulation or iron overload, has been reported in animal models, suggesting the potential involvement of ferroptosis in RP (8). Copper is also an essential trace that plays an important role in the structure and physiology of retina (36). Previous studies have tried to explore the relationship between copper metabolism and RP, but came out with contradictory results. In a study involving 15 primary RP patients from India, Gahlot et al. described the changes in copper metabolism including: (i) normal or slightly lower serum copper level, (ii) notably reduced ceruloplasmin concentration, and (iii) high urinary copper excretion (9). These finding serve as a sign of chronic copper toxicity. Moreover, liver biopsies from some RP patients suggested slight, non-specific changes, which indicated that copper metabolism in primary RP may also be altered. Rao et al. also reported that copper metabolism changed in RP cases, but in different parameters, and the degree were not comparable in the severity to those of WD patients (37). On the contrary, the serum copper levels revealed by Karcioglu et al. were in an opposite tendency to that of Gahlot's study (38). Other results by Marmor et al. (10), Ehlers et al. (39), and Atmaca et al. (40) also did not support the existence of copper metabolic abnormalities in individuals with RP. Thus, hypothesis was proposed that exogenous factors such as diet, overall nutrition and genetic isolation may account for the discrepancies between the normal results of copper metabolism and the striking Indian findings (10). By far, there is still lack of convincing evidence to indicate that RP is related to abnormal copper metabolism (Supplementary Table 2).

## Conclusion

**Figure 4:**
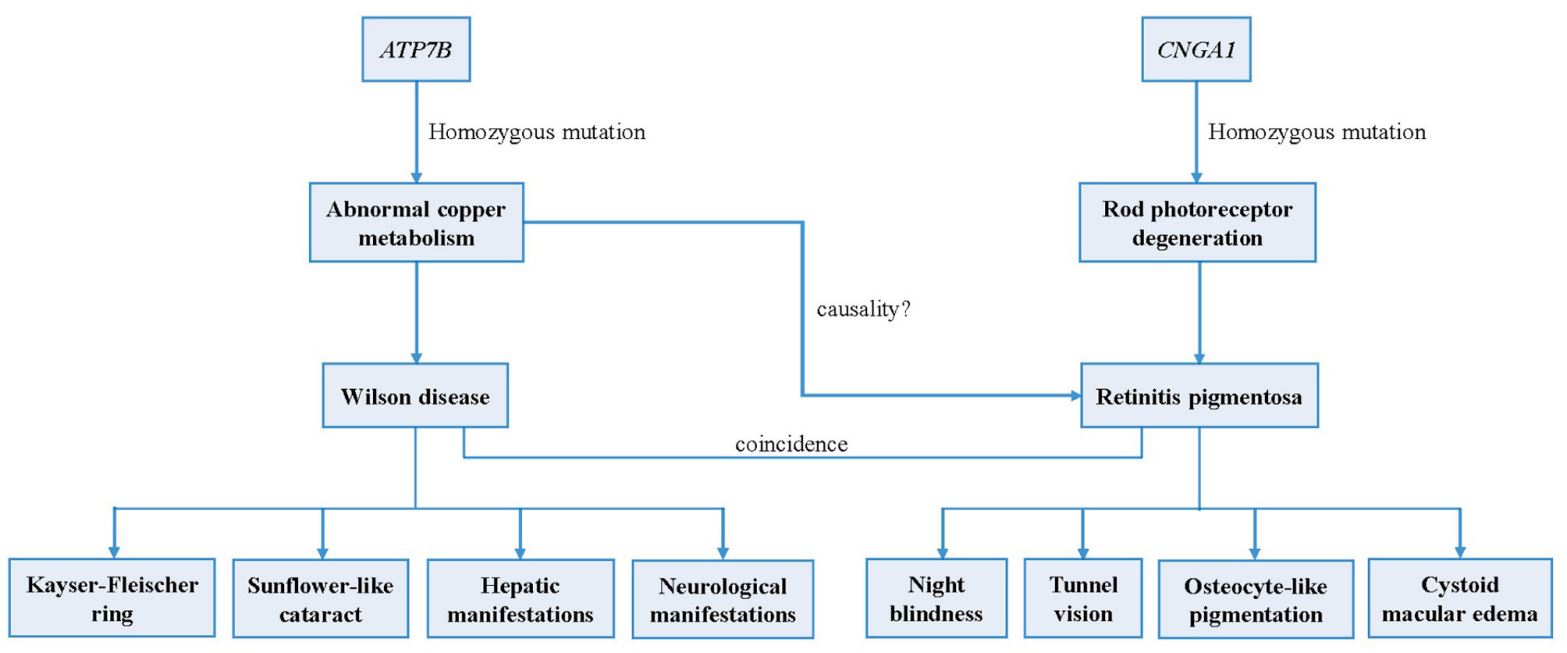
Logical mapping illustrates the possible pathogenesis of Wilson disease and retinitis pigmentosa in this patient.

In summary, a case of concurrent WD and binocular RP was reported for the first time. We identified a deleterious homozygous mutation in the patient's *ATP7B* gene that caused WD, as well as a homozygous mutation in *CNGA1* that conformed to gene co-segregation that potentially led to RP (Figure 4). Since there remains no sufficient evidence to support that the occurrence of RP is associated with WD or abnormal copper metabolism, we speculate that the two pathogenic gene mutations lead to the coincidence of the two genetic disorders, respectively.

## Data Availability Statement

The original contributions presented in the study are included in the article/Supplementary Materials, further inquiries can be directed to the corresponding author/s.

## Ethics Statement

The studies involving human participants were reviewed and approved by Ethics Committee of the Second Affiliated Hospital of Zhejiang University. The patients/participants provided their written informed consent to participate in this study. Written informed consent was obtained from the individual(s) for the publication of any potentially identifiable images or data included in this article.

## Author Contributions

ZY wrote the first draft. XJ guided the genetic analysis and revised the manuscript. XL and QZ recorded the medical information. KW and MC contributed to the treatment of the patient and made revisions of the manuscript. All authors contributed to the critical revision and provided final approval of the submitted version of this article.

## Funding

This study was supported by National Natural Science Foundation of China (No. 82171045).

## Conflict of Interest

The authors declare that the research was conducted in the absence of any commercial or financial relationships that could be construed as a potential conflict of interest.

## Publisher's Note

All claims expressed in this article are solely those of the authors and do not necessarily represent those of their affiliated organizations, or those of the publisher, the editors and the reviewers. Any product that may be evaluated in this article, or claim that may be made by its manufacturer, is not guaranteed or endorsed by the publisher.

## Footnotes

^1^RetNet: Summaries (uth.edu).

^2^ https://www.ncbi.nlm.nih.gov/clinvar/variation/3852/

^3^ http://swissmodel.expasy.org/

^4^RetNet: Summaries (uth.edu).
