# Supplementary material for: Case Report: The First Reported Concurrence of Wilson Disease and Bilateral Retinitis Pigmentosa
Source: Front Med (Lausanne). 2022 Apr 28;9:877752. doi: 10.3389/fmed.2022.877752 (PMC9098211; doi:10.3389/fmed.2022.877752)
Supplement: Supplementary file 1 [file Data_Sheet_1.docx]

Supplementary Material

**Supplementary Table 1.** Identification of the patient’s genetic mutations and variants.

| **Gene name** | **GenBank ID: Exon: Nucleotide change: Amino Acid change** | **State** | **Variation type** | **SNP ID** | **Inheritance pattern** | **OMIM phenotype** | **ACMG grade** |
| --- | --- | --- | --- | --- | --- | --- | --- |
| *ATP7B* | NM_000053:exon8:c.G2333T:p.R778L | Homozygous | Missense | rs28942074 | AR | WD | Pathogenic |
| *CNGA1* | NM_0011422564:exon5:c.C453A:p.Y151X | Homozygous | Nonsense | / | AR | RP49 | Pathogenic |
| *RP2* | NM_006915:exon2:c.T248C:p.I83T | Hetorozygous | Missense | rs782580817 | XL | RP2 | Uncertain significance |
| *SNRNP200* | NM_014014:exon15:c.C1898T:p.A633V | Hetorozygous | Missense | rs749655800 | AD | RP33 | Uncertain significance |

AR: autosomal recessive; XL: X-linked; AD: autosomal dominant; WD: Wilson disease; RP: retinitis pigmentosa; SNP: single nucleotide polymorphism.

**Supplementary Table 2.** Summary of findings between copper metabolism and retinitis pigmentosa.

| **No.** | **First author** | **Publication year** | **Sample size** | **Age range** | **Copper concentration（μg/100ml）** | | **Ceruloplasmin concentration（/100ml）** | | **urinary excretion of copper （μg/24h）** | | **Conclusion** | **Reference** |
| --- | --- | --- | --- | --- | --- | --- | --- | --- | --- | --- | --- | --- |
|  |  |  |  |  | **Control** | **RP** | **Control** | **RP** | **Control** | **RP** |  |  |
| 1 | Gahlot DK | 1976 | 15 | 10-42 | 97.7 ± 27.1 | 77.6±22.9 | 20.1 mg | 7.0 mg↓ | 32.0 ± 14.0 | 186.1 ± 54.3↑ | Relevant | Ref 8 |
| 2 | Ehlers N | 1977 | 15 | 10-62 | NA | NA | NA | NA | NA | NA | Irrelevant | Ref 30 |
| 3 | Marmor MF | 1978 | 38 | 8-66 | 113 ± 16 | 104 ± 19 | 36.4 ± 5.5 μg | 35.3 ± 7.3 μg | NA | NA | Irrelevant | Ref 9 |
| 4 | Rao SS | 1981 | 24 | 12-38 | 102.4 | 135.7 | 30.7 mg | 22.8 mg↓ | 35.7 | 79.5↑ | Relevant | Ref 28 |
| 5 | Karcioglu ZA | 1984 | 26 | NA | NA | 126.1 ± 34.8↑ | NA | NA | NA | NA | Relevant | Ref 29 |
| 6 | Atmaca LS | 1989 | 83 | 11-71 | 121.7±10.3 | 113.3 ± 3.7 | NA | NA | NA | NA | Irrelevant | Ref 31 |

RP: retinitis pigmentosa; NA: not available; Ref: reference; ↑: increase; ↓: reduce.


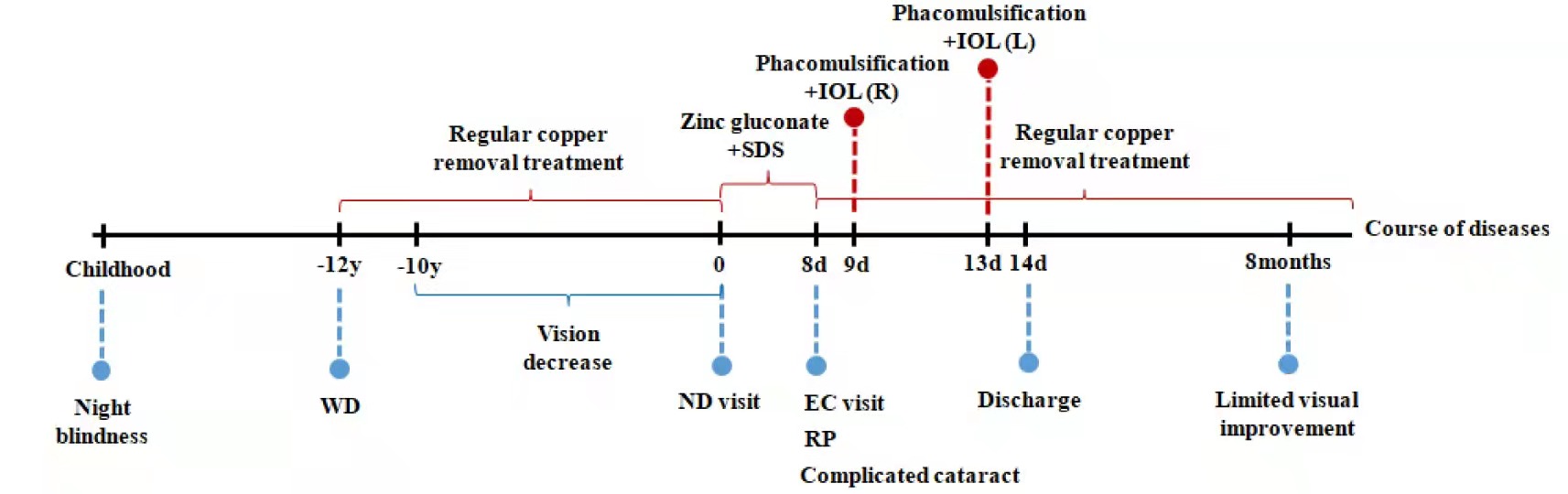


**Supplementary Figure 1.** Timeline showcasing the course of diseases and relevant treatment. Abbreviations: WD: Wilson disease; ND: neurology department; EC: eye center; RP: retinitis pigmentosa; SDS: sodium dimercaptopropane sulfonate; IOL: implant intraocular lens; R: right eye, L: left eye.
